# Loss of dynamin 1-like protein impairs mitochondrial function and self-renewal, and activates the integrated stress response in human embryonic stem cells

**DOI:** 10.3389/fgene.2025.1628178

**Published:** 2025-07-28

**Authors:** Artur Cieslar-Pobuda, Safak Caglayan

**Affiliations:** ^1^ Centre for Molecular Medicine Norway, University of Oslo and Oslo University Hospital, Oslo, Norway; ^2^ Department of Cancer Immunology, Institute of Cancer Research, Oslo University Hospital, Oslo, Norway; ^3^ Division of Mental Health and Substance Abuse, University Hospital of North Norway, Tromsø, Norway

**Keywords:** human embryonic stem cells, DNM1L, DRP1, mitochondrial fission, mitochondrial dysfunction, integrated stress response, self-renewal, pluripotency

## Abstract

Dynamin 1-like protein (DNM1L/DRP1) is a crucial regulator of mitochondrial fission in cells and pathogenic mutations in DNM1L are linked to developmental and metabolic disorders in humans. While the role of DNM1L has been described in patient-derived fibroblasts, its function in early human development remains unclear. In this study, we generated DNM1L deficient human embryonic stem cells (hESCs) using CRISPR/Cas9 to investigate the consequences of DNM1L deficiency and impaired mitochondrial fission on stem cell function. *DNM1L* ^−/−^ hESCs exhibited hyperfused mitochondrial networks, reduced mitochondrial membrane potential, and elevated oxidative stress, indicating compromised mitochondrial fitness. Functionally, *DNM1L* ^−/−^ hESCs showed diminished self-renewal, and reduced expression of the core pluripotency factor OCT4, while NANOG expression was unaffected. We further found that differentiation potential toward the early ectodermal lineage was impaired, whereas early endodermal and mesodermal differentiation remained intact. Notably, integrated stress response (ISR) pathway was activated in *DNM1L* ^−/−^ hESCs, as shown by increased phosphorylated eIF2a and upregulation of downstream targets including activating transcription factor 4 (ATF4), ATF3, ATF5, and DDIT3. Restoring DNM1L expression by reintroduction of DNM1L into the *AAVS1* locus rescued mitochondrial morphology and function, normalized ISR activation, and restored self-renewal and OCT4 expression in *DNM1L* ^−/−^ hESCs. These findings demonstrate that DNM1L is essential for maintaining mitochondrial homeostasis, stress response, self-renewal, and pluripotency in hESCs, and emphasize the importance of mitochondrial fission in stem cell function.

## 1 Introduction

Pluripotent stem cells (PSCs) have an unlimited self-renewal potential without losing their identity while maintaining their pluripotency, the potential to differentiate into any cell type. Mitochondria play an essential role in function of stem cells by integrating environmental signals to cellular energy metabolism (Zhang, Menzies, and Auwerx, 2018). In addition, metabolites and by-products of energy metabolism are involved in retrograde signaling to regulate various cellular functions (Zhang, Menzies, and Auwerx, 2018). Mitochondrial homeostasis is correlated with the function and regulation of various cellular processes including energy metabolism, cell fate, and apoptosis (Kasahara and Scorrano, 2014; Chakrabarty and Chandel, 2021; Jasra et al., 2023). Mitochondrial morphology and dynamics are regulated by mitochondrial fission and fusion, processes that are controlled by orchestrated action of several mitochondrial and cytoplasmic proteins. Membrane tethered mitofusin 1 (MFN1) and MFN2 are involved in fusion of the outer mitochondrial membranes while optic atrophy 1 (OPA1) is required for fusion of the inner mitochondrial membranes. Cytoplasmic dynamin-1-like protein (DNM1L/DRP1) mediates fission of mitochondria.

DNM1L is essential for development. Deletion of *Dnm1l* causes embryonic lethality in mice (Ishihara et al., 2009; Wakabayashi et al., 2009), and embryos deficient for Dnm1l have a significantly smaller body size and poorly developed organs (Ishihara et al., 2009). In humans, bi-allelic loss-of-function or dominant-negative *DNM1L* mutations cause fatal developmental and metabolic disorders (Fahrner et al., 2016; Waterham et al., 2007). Studies using fibroblasts derived from patients with *DNM1L* mutations showed hyperfused mitochondrial networks confirming the role of DNM1L in mitochondrial fission (Whitley et al., 2018; Nasca et al., 2016). However, the role of DNM1L and mitochondrial fission in human embryonic stem cells (hESCs) remains unclear. Addressing this knowledge gap might provide insights into how DNM1L regulates early human development, pluripotent stem cell function, and the pathogenesis of DNM1L-linked diseases. To this end, we generated DNM1L deficient hESCs to characterize the effects of DNM1L on mitochondrial function, self-renewal, and pluripotency in hESCs.

## 2 Materials and methods

### 2.1 hESC culture

The cell lines present in this study were obtained from the Wisconsin Stem Cell Bank (WiCell, Madison, United States). Female WA#22 hESCs were maintained on mitomycin inactivated mouse embryonic fibroblast (MEF) layers. hESC media contained DMEM/F12, 15% fetal bovine serum qualified for hESCs (Biological Industries), 5% KnockOut Serum replacement, 1x non-essential amino acids, 1x Glutamax, 50 µM Beta-mercaptoethanol (all from Thermo Fisher Scientific) supplemented with 5 ng/mL basic FGF (Miltenyi). The use of this media composition and these reagents was optimized and validated for culturing and maintaining hESCs in our previous studies (Caglayan et al., 2020; Cieslar-Pobuda et al., 2020). hESCs were split when colonies became too dense or too large. To passage hESCs, cells were treated with 1 mg/mL Collagenase (Thermo Fisher Scientific). Colonies were collected using hESC media, gently pipetted with fresh media while avoiding the formation of single-cell suspensions, then centrifuged, and resuspended in fresh media before being transferred to MEF-coated plates.

For experiments in which hESCs were cultured under feeder-free conditions, Matrigel-coated plates (Corning Matrigel hESC-qualified matrix; #354277) were used. Matrigel was diluted in precooled DMEM/F12 media according to the batch-specific dilution factor provided by the manufacturer and as described in a previous study (Fang and Li, 2021). To maintain hESCs in feeder-free conditions, cells were cultured in MEF-conditioned media. MEFs were incubated with hESC media overnight, and the conditioned media was collected the following day. Before use, MEF-conditioned media was supplemented with 20 ng/mL basic FGF to support hESC growth, as described in the following application protocol: https://www.thermofisher.com/no/en/home/references/protocols/cell-culture/stem-cell-protocols/ipsc-protocols/culture-hescs-conditioned-medium.html.

### 2.2 Generation of genetically modified hESCs

Targeted deletion of the *DNM1L* gene was achieved using CRISPR-Cas9 using the single guide RNAs (sgRNAs) listed in Supplementary Table S1. Human codon optimized SpCas9-expressing px330-U6-Chimeric_BB-CBh-hSpCas9 plasmid is a gift from Feng Zhang (Addgene plasmid # 42230). Oligonucleotides were designed using the http://crispr.mit.edu/webtool, and annealed oligos were cloned into the px330 plasmid according to the published protocol (Cong et al., 2013).

Prior to electroporation, hESCs were cultured in hESC media supplemented with 10 µM Rho Kinase (ROCK)-inhibitor (Y-27632; ATCC) for 24 h to improve survival and recovery of dissociated cells (Watanabe et al., 2007; Baharvand et al., 2010). hESC colonies were harvested with 1 mg/mL collagenase and single cell suspension was prepared with Trypsin (Life Technologies). Single cell hESCs were resuspended in 500 µL Optimem (Life technologies) and electroporated with 15 μg of each CRISPR plasmid (200 V, 500μF, 0.4 cm cuvettes). Subsequently, cells were plated on MEF feeder layers in hESC medium supplemented with 10 μM ROCK-inhibitor. Between 10–14 days after electroporation, individual colonies were picked, and *DNM1L* deficiency was assessed by PCR analysis. Positive clones were confirmed by Sanger sequencing and correctly targeted clones were expanded.

For rescue cells, the *DNM1L* cDNA was amplified from the mCh-Drp1 plasmid (a gift from Gia Voeltz (Addgene plasmid #49152)) without mCherry tag and cloned into the pAAVS1-Nst-CAG-DEST vector as described (Oceguera-Yanez et al., 2016), and verified by Sanger sequencing. To generate DNM1L rescue hECSs (*DNM1L* ^−/−^; AAVS1-*DNM1L*), plasmid DNAs targeting the AAVS1 locus were delivered to *DNM1L* ^−/−^ hESCs by electroporation. Cells were selected with G418, and colonies were picked, expanded, and genotyped as described (Oceguera-Yanez et al., 2016). Heterozygously targeted DNM1L rescue hESCs (*DNM1L* ^−/−^; AAVS1-*DNM1L* ^*+/−*^), which were identified by PCR analysis using DNA183, DNA803, and DNA804 primers and verified by qPCR, were used in all experiments.

### 2.3 Genotyping

WA#22 cells were lysed in 250 µL DNA lysis buffer (50 mM Tris pH8.0, 5 mM EDTA pH8.0, 1% SDS, 150 mM NaCl, 200 μg/mL proteinase K) overnight at 55°C. DNA was precipitated using 100% isopropanol and washed with 70% ethanol. Genomic DNA was resuspended in nuclease-free dH2O (Life technologies) and 50 ng of genomic DNA was used for PCR analysis. Primers FW 5′-GCA ATG CCA GAA ACC ATA TAC-3′ and RV 5′-GCT ACA GTG CTG AAT GAT CCT-3′ were used to detect deletion of exon 10.

### 2.4 DNA and RNA analysis

Measuring the ratio between mitochondrial and nuclear DNA content is used to quantify mitochondrial DNA copy number (Rooney et al., 2015). DNA samples were isolated from hESCs using the DNeasy Blood & Tissue kit (Qiagen) according to the manufacturer’s instructions. DNA concentration was measured using Nanodrop instrument. To measure mitochondrial and nuclear DNA content we performed real-time PCR in duplicates using 10 ng DNA, 1 x SYBR green and 500 nM primers for each reaction. We calculated mitochondrial: nuclear DNA copy number ratio by normalization of CT values that were obtained from amplification curves of mitochondrial NADH-ubiquinone oxidoreductase chain 1 (MT-ND1) and MT-ND4 genes, and nuclear beta-2-microglobulin (B2M) gene.

RNA was isolated using Qiazol or the RNAeasy kit (both Qiagen) according to the manufacturer’s instructions. ABI cDNA synthesis kit was used to transcribe 1 µg of RNA into cDNA. QRT-PCR reactions were prepared with 10 ng of cDNA, 1x SYBR green, 500 nM primers. Samples were run as technical replicates using the ABI9600 or BioRAD CFX96 instrument. Specificity of amplification was determined using melt curve analysis. Expression was calculated based on the Delta-Delta-CT method and expression was normalized to the housekeeping gene 18S rRNA and parental control cells. Primer sequences are listed in Supplementary Table S1.

### 2.5 Protein extraction and immunoblotting

Total cell lysates using lysis buffer (10 mM Tris pH7.5, 150 mM NaCl, 1 mM EDTA, 1% SDS, 0.5% Na-deoxycholate) with protease inhibitor (Thermo Fisher Scientific) were sonicated at 4°C to shear DNA. Protein concentrations were measured using the BCA protein assay kit (Thermo Fisher Scientific) and equal amounts of proteins were loaded on 4%–20% gradient gels (Bio-Rad) in SDS running buffer (25 mM Tris-base, 192 mM glycine, 0.1% SDS). Proteins were transferred to immobilon-P PVDF membranes in transfer buffer (25 mM Tris-base, 192 mM glycine, 10% methanol). Membranes were blocked with 5% milk/0.1% Tween-20 in TBS buffer (25 mM Tris-base, 137 mM NaCl, 2.7mM KCl, pH 7.4) and incubated overnight with primary antibodies (1 μg/mL) at 4°C. Respective HRP-linked secondary antibodies (Jackson ImmunoResearch) were used at 1 μg/mL concentration in blocking buffer for 1 h at room temperature. Beta-actin was used as a loading control in immunoblotting experiments. Chemiluminescent imaging was performed using the Bio-Rad Imaging system and quantification was performed using Bio-Rad software. Antibodies are listed in Supplementary Table S2.

### 2.6 Analysis of colony growth and live cell automated imaging

To monitor colony growth, we harvested hESC colonies using 1 mg/mL collagenase. We took phase-contrast images of hESC colonies 5 days after passaging using the Zeiss Axio Vert.A1 microscope equipped with an AxioCam MRm camera. We analyzed colony growth by measuring diameter of each hESC colony using the Zeiss Zen software Blue edition.

Single cell suspensions of hESCs were prepared using Accutase (Thermo Fisher Scientific). An equal number of hESCs were seeded on Matrigel (Corning) coated 24-well tissue-culture plates (Falcon) in MEF-conditioned hESC media supplemented with 20 ng/mL basic FGF and 10 µM ROCK inhibitor (Y-27632; ATCC) to improve survival and recovery of dissociated cells. Fresh MEF-conditioned hESC media was added after 24 h and plates were placed in a humidified tissue culture incubator equipped with an IncuCyte Live Cell Analysis System.

### 2.7 Analysis of mitochondrial morphology

Single cell suspensions of hESCs were plated on Matrigel coated glass cover slips in 24-well tissue-culture plates (Falcon) using MEF-conditioned hESC media supplemented with 20 ng/mL basic FGF and 10 µM ROCK-inhibitor. After 24 h, cells were incubated with 2 µL of Cell light mitochondria-GFP, BacMam 2.0 (mito-GFP) reagent per 10.000 cells overnight according to the manufacturer’s instructions. Next, hESCs were fixed with 2% methanol-free formaldehyde (Thermo Fisher Scientific) for 15 min, washed with PBS and mounted on coverslips using ProLong Diamond antifade mountant (Thermo Fisher Scientific). Photographs were taken with Zeiss LSM 800 confocal laser scanning microscope. Images were processed using the Zeiss Zen software Blue edition.

### 2.8 Analysis of oxygen consumption rate

We used the Seahorse XF Cell Mito Stress Test and XFe96 Analyzer (Agilent) to measure oxygen consumption rate (OCR). 20,000 hESCs were seeded onto a matrigel-coated XF96 cell culture microplate (Agilent) and incubated overnight in hESC medium supplemented with 10 µM ROCK inhibitor. One hour before the analysis, culture medium was changed to Seahorse XF DMEM Medium, pH 7.4 (Agilent) supplemented with 2 mM glutamine, 1 mM pyruvate and 10 mM glucose. Selective inhibitors of mitochondrial electron transport chain: Oligomycin (1 µM), carbonyl cyanide p-(trifluoromethoxy) phenylhydrazone (FCCP) (2 μM), and rotenone/antimycin A (0.5 μM) were injected sequentially during the measurements at indicated time points. Basal respiration was determined as OCR before injection of oligomycin minus OCR after injection of rotenone/antimycin A. ATP-linked respiration was determined as OCR before oligomycin injection minus OCR after oligomycin treatment. Maximal respiration was determined as the OCR after FCCP minus non-mitochondrial OCR (OCR after rotenone/antimycin A treatment). After the assays, hESCs in the assay plates were lysed using lysis buffer (10 mM Tris pH7.5, 150 mM NaCl, 1 mM EDTA, 1% SDS, 0.5% Na-deoxycholate) supplemented with protease inhibitors. Protein concentrations were determined using the BCA Protein Assay Kit (Thermo Fisher Scientific), and oxygen consumption and extracellular acidification rates measured in each well were normalized to protein concentration in the corresponding well.

### 2.9 Immunostaining

Cells were fixed with 2% methanol-free formaldehyde (Thermo Fisher Scientific) for 15 min and permeabilized with 0.3% Triton X-100 in PBS for 10 min at room temperature. Cells were incubated in blocking solution (1% BSA/2% FBS/0.1% Tween in PBS) for 30 min at room temperature followed by primary antibody (5–8 μg/mL in PBS) incubation overnight at 4°C. Samples were washed with PBS and incubated with 5 µg/mL of the respective Alexa-488 or -546 conjugated secondary antibodies (Thermo Fisher Scientific) for 1 h at room temperature. Nuclei were stained with DAPI. Samples were mounted with ProLong Diamond antifade mountant (Thermo Fisher Scientific). Images were taken with Zeiss Axio Vert.A1 microscope equipped with an AxioCam MRm camera or Zeiss LSM 800 confocal laser-scanning microscope. Images were processed using the Zeiss Zen software Blue edition. Antibodies are listed in Supplementary Table S2.

### 2.10 Phosphoarrays

Human Phosphorylation Pathway Profiling Array C55 (RayBiotech) was used to assess the relative levels of phosphorylation of 55 proteins in 5 well-known signaling pathways level of: MAPK, AKT, JAK/STAT, NF-κB, and TGFβ. Cells were lysed in Lysis Buffer supplemented with Phosphatase and Protease Inhibitor Cocktails according to manufacturer’s instructions. Protein concentrations were measured using the BCA protein assay kit (Thermo Fisher Scientific) and equal amounts of proteins (500 µg) were loaded onto each membrane with printed primary antibodies. After incubation with secondary HRP antibodes and subsequent washing steps, chemiluminescent imaging was performed using the Bio-Rad Imaging system. Signal quantification was performed using ImageJ software.

### 2.11 Flow cytometry analysis

We used MitoProbe TMRM assay kit (#M20036 from ThermoFisher Scientific) to measure mitochondrial membrane potential in hESCs. We prepared single cell suspensions of hESCs using accutase (Life Technologies) and analyzed mitochondrial membrane potential according to the manufacturer’s instructions. To induce mitochondrial depolarization, hESCs were incubated with 50 µM carbonyl cyanide m-chlorophenyl hydrazine (CCCP) for 5 min in a tissue culture incubator, according to the manufacturer’s instructions. hESCs that were incubated without CCCP (baseline) and with CCCP were then incubated with 20 nM tetramethylrhodamine methyl ester (TMRM) for 30 min in a tissue culture incubator. Cells were washed once with 1 mL PBS and immediately analyzed using BD FACSCanto flow cytometer. Kaluza software (Beckman Coulter) was used for analysis and illustration of flow cytometer data.

Levels of reactive oxygen species (ROS) were measured using general sensitive oxidative stress indicator 5-(and 6-) chloromethyl-2′,7′-dichlorodihydrofluorescein diacetate, acetyl ester (CM-H2DCFDA), which is a widely used indicator of intracellular ROS (Canli et al., 2017; Zhou et al., 2016). Labeling and measurement of ROS were performed according to the manufacturer’s instructions (Cat# C6827, Thermo Fisher Scientific). Single cell suspensions were loaded with 2 µM CM-H2DCFDA for 20 min in a tissue culture incubator. After washing with PBS, cells were analyzed immediately using BD FACSCanto flow cytometer. Unlabeled hESCs were used as negative control and hESCs that were pre-incubated with 0.5 mM H_2_O_2_ were used as positive control (data not presented). Data was analyzed using Kaluza software (Beckman Coulter) and mean fluorescence intensity values were presented.

### 2.12 Statistical analysis

Visualization and statistical analysis of data were performed using GraphPad Prism. Data were presented as mean ± standard error of the mean (SEM). Student’s t-test and analysis of variance (ANOVA) with Sidak *post hoc* test were used to analyze differences between groups. P < 0.05 was considered as statistically significant difference. *p < 0.05, **p < 0.01, ***p < 0.001.

## 3 Results

**FIGURE 1:**
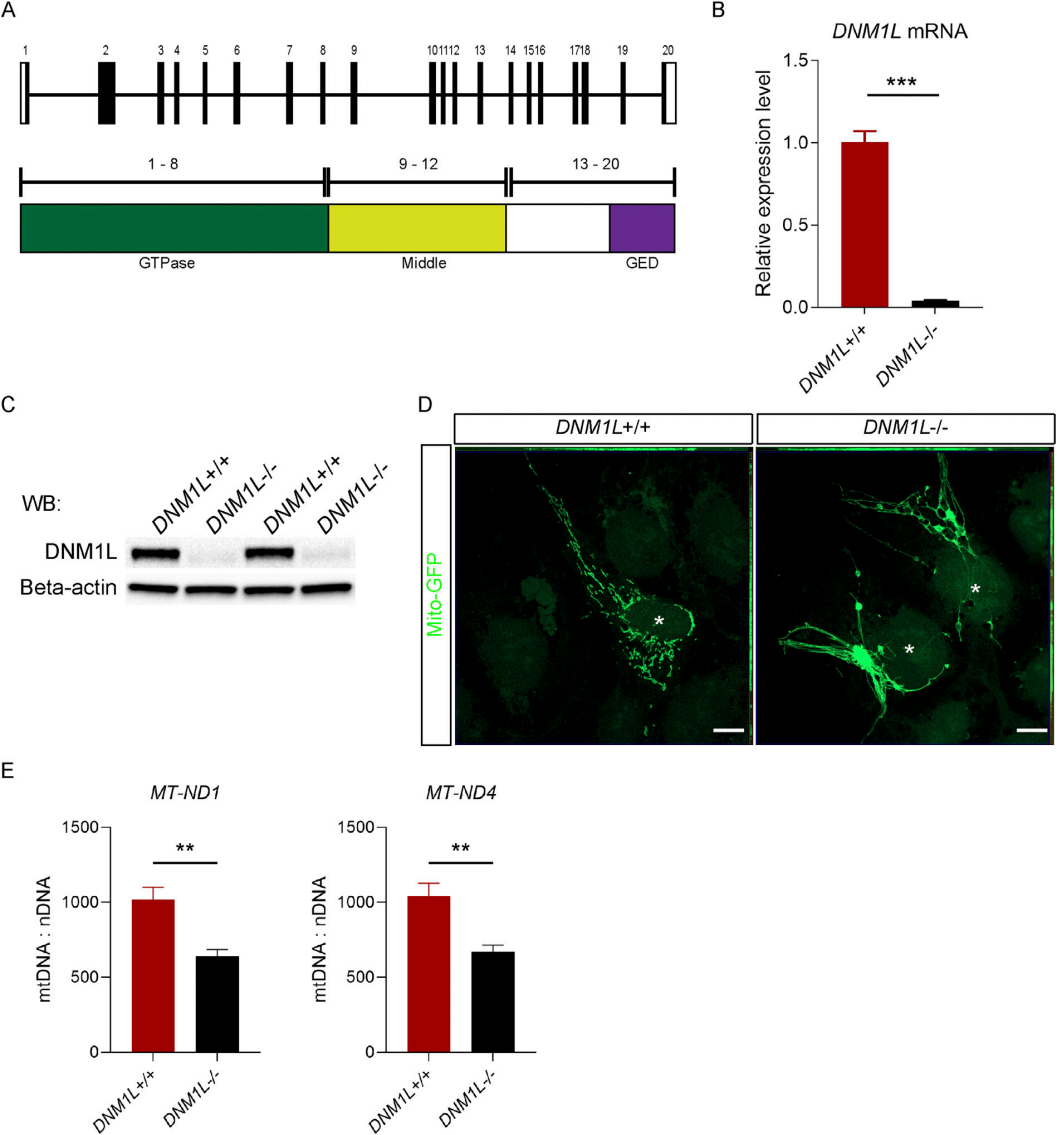
Loss of DNM1L impairs mitochondrial structure and DNA content in hESCs. **(A)** Schematics of the genomic structure and corresponding protein domains of *DNM1L*. **(B)** QRT-PCR analysis of *DNM1L* mRNA expression in *DNM1L* ^+/+^ and *DNM1L* ^−/−^ hESCs. **(C)** Analysis of DNM1L protein expression in *DNM1L* ^+/+^ and *DNM1L* ^−/−^ hESCs by immunoblotting. Beta-actin is used as loading control. **(D)** Representative confocal microscopy pictures of *DNM1L* ^+/+^ and *DNM1L* ^−/−^ hESCs transduced with mito-GFP. Scale bar, 10 µM. **(E)** Mitochondrial DNA content in *DNM1L* ^+/+^ and *DNM1L* ^−/−^ hESCs. Copy number of mitochondrial *ND1* and *ND4* genes are measured using qPCR, and normalized to nuclear *B2M* gene. N = 3 experiments. Student’s t-test is used to analyze differences between two groups. *p < 0.05, **p < 0.01, ***p < 0.001.

Using CRISPR/Cas9 we deleted part of exon 10 in *DNM1L* gene (Figure 1A), which results in a frameshift and premature stop codon downstream of exon 10. Quantitative reverse transcription polymerase chain reaction (qRT-PCR) and Western blot analyses showed that DNM1L mRNA (Figure 1B) and protein (Figure 1C) were absent in targeted hESCs. Since knockout of DNM1L is known to cause fission defects (Smirnova et al., 2001), we first assessed mitochondrial morphology in *DNM1L* ^−/−^ hESCs. Confocal microscopy analysis of hESCs transduced with mito-GFP encoding vectors showed that *DNM1L* ^−/−^ hESCs in contrast to parental hESCs exhibited an elongated and hyperfused mitochondrial network forming mesh-like structures (Figure 1D). When we quantified copy numbers of mitochondrial genes encoding NADH-ubiquinone oxidoreductase chain 1 (MT-ND1) and MT-ND4, we found that *DNM1L* ^−/−^ hESCs had significantly less mitochondrial DNA content compared with parental hESCs (Figure 1E).

**FIGURE 2:**
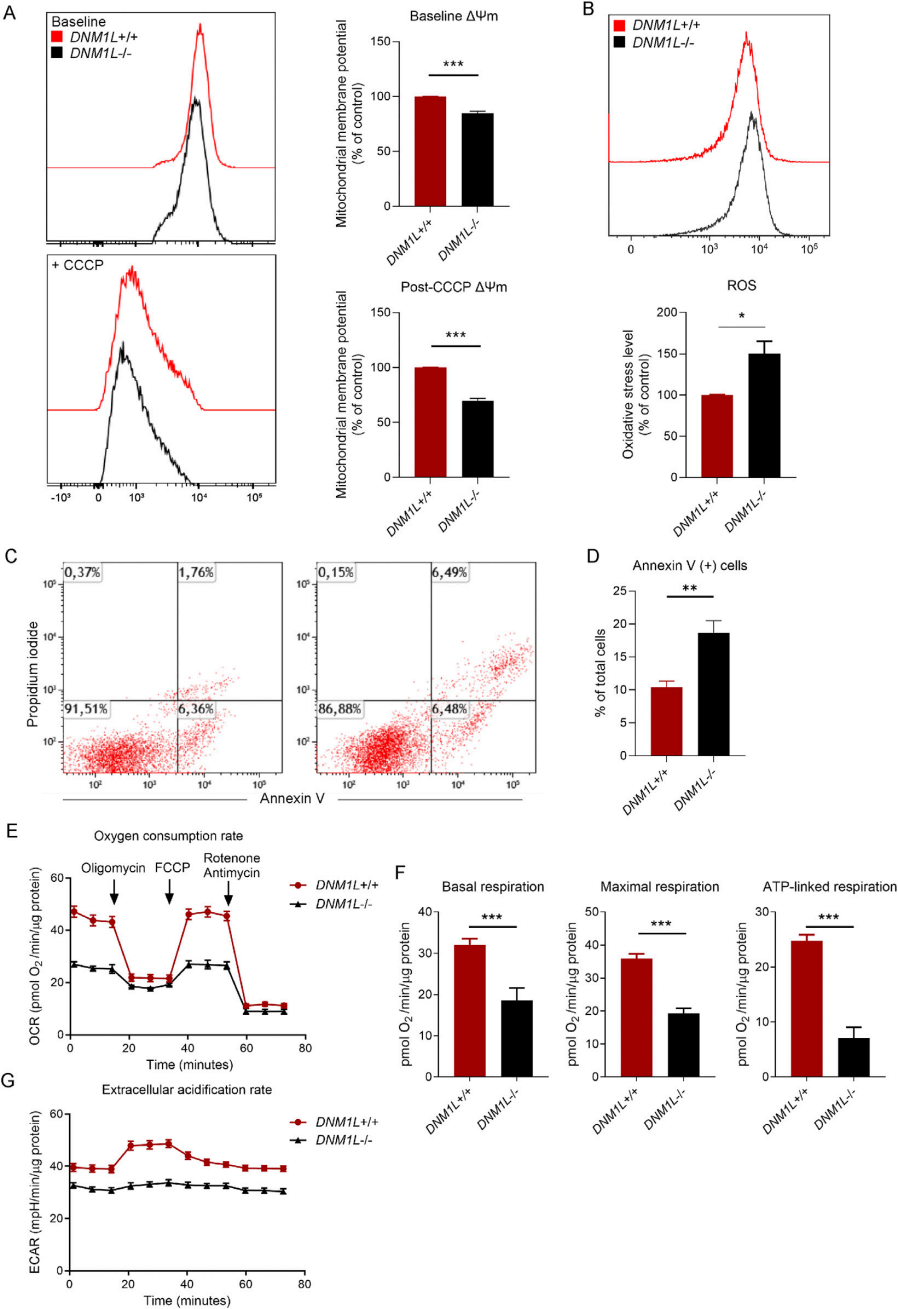
Loss of DNM1L disrupts mitochondrial function, increases oxidative stress, and promotes apoptosis in hESCs. **(A)** Left panel: Representative histograms showing flow cytometry measurements of mitochondrial membrane potential in *DNM1L* ^+/+^ and *DNM1L* ^−/−^ hESCs. CCCP is used to depolarize the mitochondrial membranes. Right panel: Quantification of flow cytometry measurements of mitochondrial membrane potential in hESCs. N = 4 experiments. **(B)** Quantification of flow cytometry measurements of reactive oxygen species (ROS) in *DNM1L* ^+/+^ and *DNM1L* ^−/−^ hESCs labeled with oxidative stress indicator CM-H2DCFDA. Mean fluorescence intensity values are normalized to the values of labeled *DNM1L* ^*+/+*^ hESCs. N = 4 experiments. **(C)** Representative flow cytometry analysis of *DNM1L* ^+/+^ and *DNM1L* ^−/−^ hESCs labeled with the early apoptosis indicator Annexin V and propidium iodide. N = 5 experiments. **(D)** Quantification of flow cytometry measurements of basal level of apoptosis in *DNM1L* ^+/+^ and *DNM1L* ^−/−^ hESCs. Mean ± SEM is shown. Student’s t-test is used to analyze difference between two groups. *p < 0.05, **p < 0.01, ***p < 0.001. **(E)** Oxygen consumption rate (OCR) in *DNM1L* ^+/+^ and *DNM1L* ^−/−^ hESCs measured by Seahorse assay. N = 2 experiments. Mean ± SEM is shown. **(F)** Basal respiration, maximal respiration and ATP-linked respiration in *DNM1L* ^+/+^ and *DNM1L* ^−/−^ hESCs. N = 2 experiments. Mean ± SEM is shown. Student’s t-test is used to analyze differences between two groups. ***p < 0.001 **(G)** Extracellular acidification rates (ECAR) in *DNM1L* ^+/+^ and *DNM1L* ^−/−^ hESCs measured by Seahorse assay. N = 2 experiments. Mean ± SEM is shown.

Next, we measured the mitochondrial membrane potential in live hESCs using tetramethylrhodamine ester dye, which is a cell-permeant fluorescent dye sequestered by active mitochondria. Flow cytometry analysis of labeled hESCs showed that baseline mitochondrial membrane potential was significantly reduced in *DNM1L* ^−/−^ compared with *DNM1L* ^+/+^ hESCs (Figure 2A). Using carbonyl cyanide m-chlorophenyl hydrazine (CCCP), an inhibitor that uncouples mitochondrial membrane potential we demonstrated that *DNM1L* ^−/−^ hESCs were more sensitive to CCCP as shown by significantly decreased membrane potential compared with parental hESCs (Figure 2A). Secondly, we analyzed oxidative stress in hESCs by labeling the live cells with CM-H2DCFDA, an indicator of cellular reactive oxygen species (ROS). Flow cytometry analysis of labeled cells revealed increased ROS levels in *DNM1L* ^−/−^ hESCs (Figure 2B). To understand the consequence of the decreased mitochondrial fitness on basal apoptosis and cell survival, we performed Annexin V/propidium iodide staining in live hESCs. Flow cytometry analysis showed that 18.6% of *DNM1L* ^−/−^ hESCs were Annexin V+ pro-apoptotic/apoptotic cells compared to 10.4% of parental hESCs (Figures 2C,D). We further analyzed mitochondrial energy metabolism by measuring oxygen consumption rate (OCR). DNM1L deficiency altered the overall OCR by decreasing basal respiration, maximal respiration, and ATP-linked respiration (Figures 2E,F; Supplementary Figure S1). Additionally, glycolysis was significantly reduced in *DNML1* ^−/−^ hESCs as shown by extracellular acidification rate (Figure 2G; Supplementary Figure S1).

**FIGURE 3:**
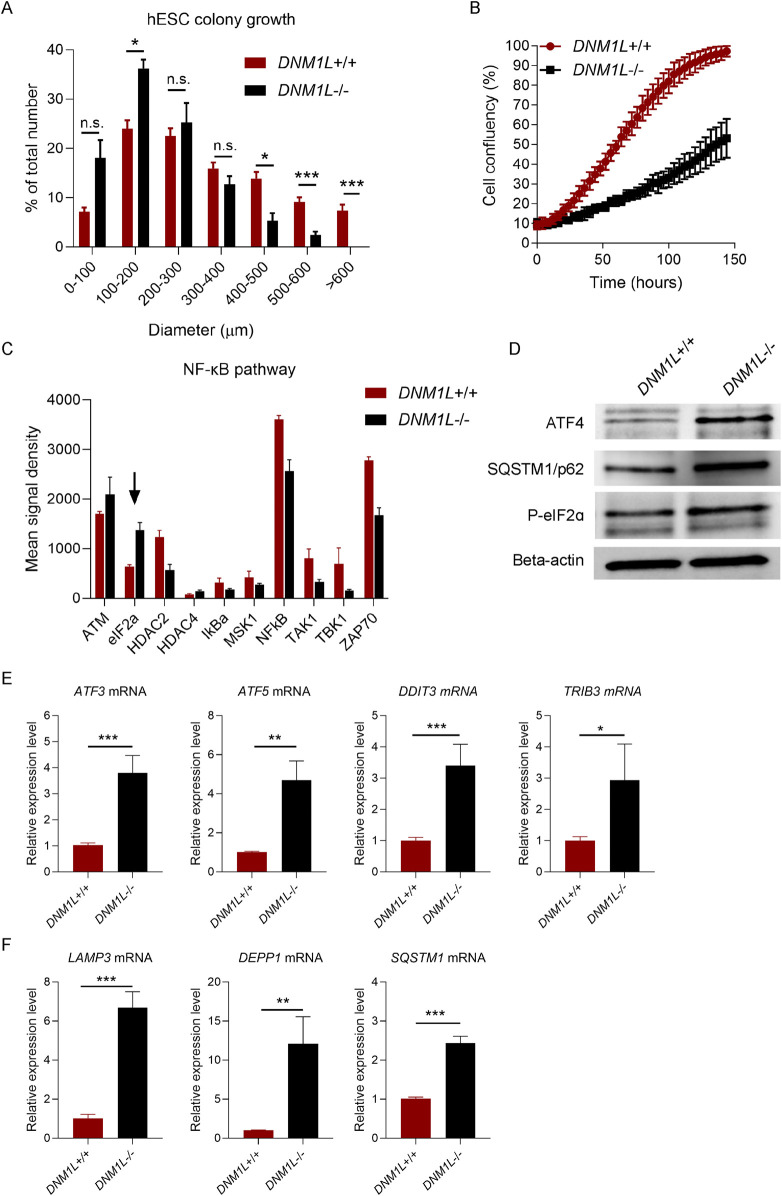
Loss of DNM1L impairs self-renewal and induces integrated stress response in hESCs. **(A)** Quantification of colony diameter after passaging of *DNM1L* ^+/+^ and *DNM1L* ^−/−^ colonies. Diameter of colonies are measured in phase-contrast images taken with bright-field microscopy. N = 5 experiments. Mean ± SEM is shown. Student’s t-test is used to analyze differences between two groups. n.s. not significant, *p < 0.05, **p < 0.01, ***p < 0.001. **(B)** Confluency of *DNM1L* ^+/+^ and *DNM1L* ^−/−^ hESCs over time assessed by Incuyte instrument. N = 4 experiments. **(C)** Densitometric analysis of immunoblots from Phosphorylation Pathway Profiling Array. **(D)** Analysis of ATF4, SQSTM1 and phospho-eIF2 alpha protein expression in *DNM1L* ^+/+^ and *DNM1L* ^−/−^ hESCs by immunoblotting. Beta-actin is used as a loading control. **(E)** QRT-PCR analysis of stress response genes *ATF3, ATF5, DDIT3, TRIB3 and* **(F)** *LAMP3, DEPP1* and *SQSTM1 (p62)* in *DNM1L* ^+/+^ and *DNM1L* ^−/−^ hESCs. N = 4 experiments. Mean ± SEM is shown. Student’s t-test is used to analyze differences between two groups. n.s. not significant, *p < 0.05, **p < 0.01, ***p < 0.001.

Inspection of colony growth in hESCs showed that *DNM1L* ^−/−^ hESC colonies were significantly smaller compared with parental hESCs (Figure 3A). When we monitored hESC expansion by time-lapse phase contrast imaging using the Incucyte automated live cell-imaging system, we found that parental hESCs were confluent after 5 days while *DNM1L* ^−/−^ hESCs reached only 50% confluency at day 5 (Figure 3B). Next, we explored signalling pathways that were potentially related with decreased self-renewal in *DNM1L* ^−/−^ hESCs. Array-based analysis of phosphorylated proteins showed that phosphorylated eIF2alpha levels were increased in *DNM1L* ^−/−^ hESCs compared to parental hESCs (Figure 3C). An increase in phosphorylated Ataxia-telangiectasia mutated (ATM) levels was also observed in *DNM1L* ^−/−^ hESCs. Immunoblotting analysis confirmed increased phosphorylated eIF2alpha levels in *DNM1L* ^−/−^ hESCs suggesting activation of integrated stress response. Furthermore, we found increased levels of activating transcription factor 4 (ATF4), which is the key regulator of integrated stress response, in *DNM1L* ^−/−^ hESCs (Figure 3D). QRT-PCR analysis verified activation of integrated stress response in *DNM1L* ^−/−^ hESCs by showing upregulated expression of several genes downstream of ATF4, including *ATF3, ATF5, DDIT/CHOP* and *TRIB3*, in *DNM1L* ^−/−^ hESCs compared with parental hESCs (Figure 3E). Autophagy is a cellular process closely related with integrated stress response and, therefore, we looked into expression of genes involved in selective autophagy in hESCs. We found that LAMP3A, DEPP autophagy regulator 1 (*DEPP1*) and sequestosome 1/ubiquitin-binding protein 62 (*SQSTM1/p62*) were significantly upregulated in *DNM1L* ^−/−^ hESCs (Figure 3F).

**FIGURE 4:**
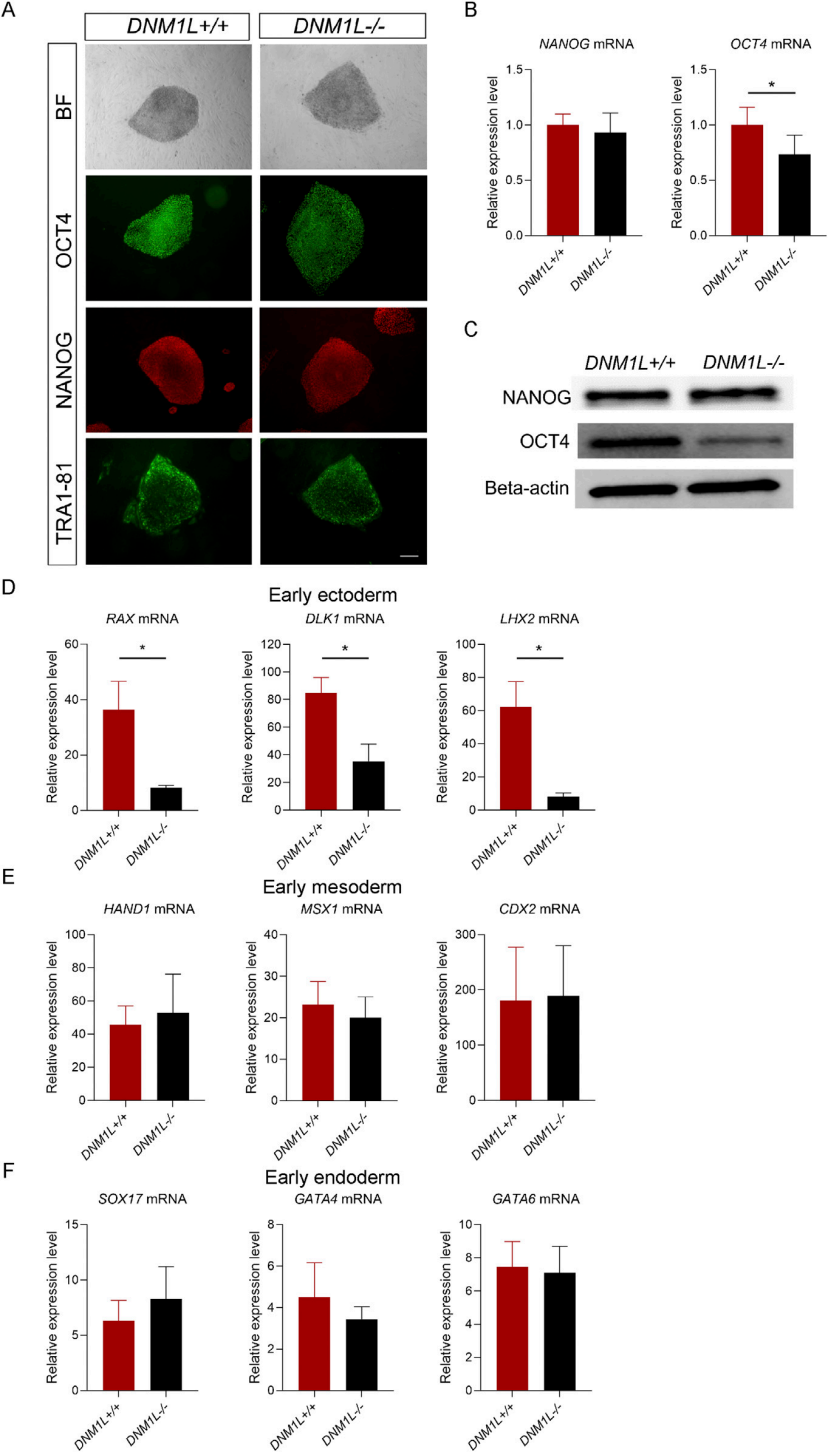
DNM1L is essential for maintenance of pluripotency and ectodermal differentiation in hESCs. **(A)** Representative bright field images and immunofluorescent staining for NANOG, OCT4 and TRA1-81 of *DNM1L* ^+/+^ and *DNM1L* ^−/−^ hESCs. Scale bar, 200 μm **(B)** QRT-PCR analysis of *NANOG* and *OCT4* mRNA expression in *DNM1L* ^+/+^ and *DNM1L* ^−/−^ hESCs. N = 4 experiments. Mean ± SEM is shown. **(C)** OCT4 and NANOG protein expression in *DNM1L* ^+/+^ and *DNM1L* ^−/−^ hESCs by immunoblotting. Beta-actin is used as a loading control. **(D)** QRT-PCR analysis of *RAX, DLK1, LHX2* mRNA expression levels in early ectoderm; **(E)** QRT-PCR analysis of *HAND1, MSX1, and CDX2* mRNA expression levels in early mesoderm and **(F)** QRT-PCR analysis of *SOX17, GATA4, GATA6* mRNA expression levels in early endoderm. In qRT-PCR analyses, gene expression is normalized to the expression of 18S control gene. N = 3 experiments. Data are presented as means ± SEM relative to *DNM1L* ^+/+^ hESCs. Student’s t-test is used to analyze differences between two groups, *p < 0.05.

Next, we assessed whether loss of DNM1L affects expression of the core pluripotency factors. Immunostaining of hESCs showed that OCT4 levels were reduced in *DNM1L* ^−/−^ hESCs compared to parental hESCs whereas NANOG and Tra1-81 levels were similar (Figure 4A). Using qRT-PCR and immunoblotting, we confirmed that NANOG mRNA and protein expression levels were comparable in *DNM1L* ^−/−^ and *DNM1L* ^+/+^ hESCs, while OCT4 mRNA and protein levels were significantly reduced in *DNM1L* ^−/−^ hESCs compared to the *DNM1L* ^+/+^ hESCs (Figures 4B,C). To study the effects of DNM1L deficiency on pluripotency of hESCs, we directed *DNM1L* ^−/−^ and parental hESCs into early ectodermal, endodermal and mesodermal germ layers. QRT-PCR analysis of differentiated cells showed that expression of transcription factors that are typically expressed in early ectoderm, including *RAX*, *DLK1*, and *LHX2* were significantly reduced in *DNM1L* ^−/−^ ectodermal cells compared to ectodermal cells derived from parental hESCs (Figure 4D). In contrast, expression of early endodermal or early mesodermal markers were similar in differentiated cells derived from *DNM1L* ^−/−^ and parental hESCs (Figures 4E,F). These results suggest that DNM1L is essential to maintain self-renewal and pluripotency of hESCs and DNM1L deficiency in hESCs results in activation of integrated stress response.

To validate that the cellular dysfunction we found in *DNM1L* ^−/−^ hESCs were directly related with the loss of DNM1L, we generated DNM1L rescue hESCs by reintroducing the *DNM1L* cDNA into the adeno-associated virus integration site 1 (*AAVS1*) locus in *DNM1L* ^−/−^ hESCs. We verified that expression of DNM1L was restored in DNM1L rescue hESCs (Supplementary Figure S2A. Immunostaining experiments showed restored DNM1L protein in DNM1L rescue hESCs (Supplementary Figure S2B). Mitochondria of DNM1L rescue hESCs were morphologically similar to parental hESCs indicating that reintroducing DNM1L was functionally competent and rescued the observed fission defect (Supplementary Figure S2C, right panel). Mitochondrial DNA content was also partially restored in DNM1L rescue cells (Supplementary Figure S2D). Furthermore, we found that DNM1L rescue hESCs had partially restored levels of mitochondrial membrane potential (Supplementary Figure S3A). ROS levels (Supplementary Figure S3B) and ratio of Annexin V+ pro-apoptotic cells (Supplementary Figure S3C) in DNM1L rescue hESCs were comparable to *DNM1L* ^+/+^ hESCs. These results suggested that mitochondrial dysfunction was rescued in DNM1L rescue hESCs. Importantly, self-renewal was restored in DNM1L rescue hESCs as shown by time-lapse phase contrast imaging (Supplementary Figure S4A). In DNM1L rescue hESCs, expression of genes involved in integrated stress response (Supplementary Figure S4B) and selective autophagy (Supplementary Figure S4C) was significantly decreased and restored to levels observed in *DNM1L* ^+/+^ hESCs. Expression of the pluripotency marker OCT4 was significantly increased in DNM1L rescue hESCs compared to *DNM1L* ^−/−^ hESCs and restored to levels similar to *DNM1L* ^+/+^ hESCs (Supplementary Figure S4D).

## 4 Discussion

In this study, we characterized the effects of DNM1L deficiency on cellular function of hESCs. The main findings in our study are 1) DNM1L deficiency in hESCs results in impaired mitochondrial function and energy metabolism, 2) Integrated stress response is activated in DNM1L deficient hESCs, and 3) DNM1L is essential for self-renewal and pluripotency of hESCs. These results are significant by showing the physiological relevance of DNM1L and mitochondrial fission in hESCs.

Dnm1l knock-out mouse embryos are smaller in size compared to wild-type mice embryos and Dnm1l deficient mouse embryonic stem cells and fibroblasts grow slower compared to the wild-type cells (Ishihara et al., 2009; Wakabayashi et al., 2009). Significance of DNM1L in development is consistent with the essential role of mitochondrial function in various cellular process including energy metabolism, autophagy, and apoptotic cell death (Kasahara and Scorrano, 2014; Chakrabarty and Chandel, 2021). Our results extend those findings about the role of mitochondria dynamics in cellular function by demonstrating that loss of DNM1L in hESCs blocked mitochondrial fission and resulted in compromised self-renewal, pluripotency, and energy metabolism.

Diminished self-renewal of DNM1L deficient hESCs might be caused by impaired mitochondrial function and decreased mitochondrial quality. Mitochondrial remodeling is important to preserve quality of mitochondria and prevent accumulation of damaged mitochondria (Twig et al., 2008). Mitochondrial distribution is coupled to cell cycle progression and depolarization of mitochondria blocks cell cycle progression in rat kidney cells (Mitra et al., 2009). Furthermore, genetic removal of mitochondrial DNA in HEK293 cells result in decreased mitochondrial membrane potential and impaired respiration rates, which in turn reduce cell proliferation (Martínez-Reyes et al., 2016). In this study, we find that DNM1L deficient hESCs have reduced mitochondrial DNA content and membrane potential. In addition, respiration rates are decreased and cellular oxidative stress is increased in DNM1L deficient hESCs suggesting inefficiency of energy metabolism and electron transport chain. Disrupted energy metabolism and inefficiency of electron transport chain might be due to altered cristae structure of mitochondria or reduced mitochondrial membrane potential which directly regulates energy metabolism (Glancy, 2020).

In response to stress, eukaryotic cells activate integrated stress response as an adaptive mechanism to restore cellular homeostasis (Zebrucka et al., 2016). Our results show that mitochondrial stress leads to a global decrease in cellular metabolism, as shown by reductions in both oxidative consumption rate and glycolysis. These results are consistent with previous studies reporting that drug-induced mitochondrial stress triggers a general decline in cellular metabolism, rather than a compensatory increase in glycolysis (Quirós et al., 2017). This global reduction may result from a diminished overall capacity of cells for protein synthesis and translation, which could be part of the cellular stress response. Thus, DNM1L deficiency and mitochondrial stress may alter the expression of proteins involved in mitochondrial fusion and energy metabolism. To elucidate these mechanisms, future studies should investigate the transcriptomic and proteomic changes associated with DNM1L deficiency in hESCs. Previous studies have shown that the integrated stress response is activated in response to mitochondrial dysfunction (Mick et al., 2020; Quirós et al., 2017; Kaspar et al., 2021). Activation of integrated stress response promotes phosphorylation of eIF2a which enhances expression of several genes, including ATF3, ATF4, ATF5, and DDIT3 (CHOP), to repair mitochondrial function and to counter metabolic stress (Ryoo, 2024). Knock-down or deletion of *Dnm1l* in mouse liver trigger activation of integrated stress response activation (Steffen et al., 2022; Wang et al., 2021). In line with those studies, we show that DNM1L deficiency in hESCs results in increased eIF2a phosphorylation and upregulation of ATF3, ATF4, ATF5, and DDIT3. Our results suggest chronic activation of the integrated stress response as a mechanism to explain cellular dysfunction in DNM1L deficient hESCs.

Our results indicate DNM1L as an important regulator of hESC differentiation. It has been shown that disruption of mitochondrial dynamics in mouse PSCs affect their *in vivo* development and impair their differentiation potential (Zhong et al., 2019). Mitochondrial dynamics might support stem cell differentiation through several pathways including energy supply, redox signaling, and epigenetic regulation via signaling molecules. Therefore, it is suggested that balance of fusion and fission events is essential for mitochondria to acquire the morphological structure needed for specific cellular functions (Lisowski et al., 2018). For example, PSCs have non-fused mitochondria and rely more on glycolysis whereas oxidative metabolism is activated during differentiation of PSCs (Lisowski et al., 2018). It is plausible that mitochondrial fission might help hESCs to adapt to changing energy demands during differentiation diminished energy supply in DNM1L deficient hESCs might interfere with progress of differentiation. Another important regulator of stem cell fate is metabolites which link energy metabolism and the chromatin landscape (Wang, Barthez, and Chen, 2024). TCA metabolites, such as acetyl-CoA, alpha-ketoglutarate, fumarate, and succinate, are substrates of chromatin modifying enzymes and regulate gene expression in somatic stem cells through epigenetic mechanisms (Chakrabarty and Chandel, 2021). Alpha-ketoglutarate has been shown as an important regulator of PSC pluripotency and differentiation (Tischler et al., 2019; Hwang et al., 2016). Altered energy metabolism and oxidative stress levels might result in changes in cellular metabolite pool. Therefore, it remains to be investigated whether metabolite levels are changed in DNM1L deficient hESCs and whether these changes are linked to epigenetic regulation required for self-renewal and proper differentiation of hESCs.

## Data Availability

The original contributions presented in the study are included in the article/Supplementary Material, further inquiries can be directed to the corresponding author.

## References

[B1] Baharvand H. Hosseini Salekdeh G. Taei A. Mollamohammadi S. (2010). An efficient and easy-to-use cryopreservation protocol for human ES and iPS cells. Nat. Protoc. 5, 588–594. 10.1038/nprot.2009.247 20203673

[B2] Caglayan S. Hashim A. Cieslar-Pobuda A. Jensen V. Behringer S. Talug B. (2020). optic atrophy 1 controls human neuronal development by preventing aberrant nuclear DNA methylation. iScience 23, 101154. 10.1016/j.isci.2020.101154 32450518 PMC7251951

[B3] Canli Ö. Nicolas A. M. Gupta J. Finkelmeier F. Goncharova O. Pesic M. (2017). Myeloid cell-derived reactive oxygen species induce epithelial mutagenesis. Cancer Cell. 32, 869–883. 10.1016/j.ccell.2017.11.004 29232557

[B4] Chakrabarty R. P. Chandel N. S. (2021). Mitochondria as signaling organelles control mammalian stem cell fate. Cell. Stem Cell. 28, 394–408. 10.1016/j.stem.2021.02.011 33667360 PMC7944920

[B5] Cieslar-Pobuda A. Ahrens T. D. Caglayan S. Behringer S. Hannibal L. Staerk J. (2020). DNMT3B deficiency alters mitochondrial biogenesis and α-ketoglutarate levels in human embryonic stem cells. STEM CELLS 38, 1409–1422. 10.1002/stem.3256 32652733

[B6] Cong L. Ann Ran F. Cox D. Lin S. Barretto R. Habib N. (2013). Multiplex genome engineering using CRISPR/cas systems. Science 339, 819–823. 10.1126/science.1231143 23287718 PMC3795411

[B7] Fahrner J. A. Liu R. Perry M. S. Klein J. Chan D. C. (2016). A novel *de novo* dominant negative mutation in DNM1L impairs mitochondrial fission and presents as childhood epileptic encephalopathy. Am. J. Med. Genet. A 170, 2002–2011. 10.1002/ajmg.a.37721 27145208 PMC5100740

[B8] Fang Y. Li X. (2021). A simple, efficient, and reliable endoderm differentiation protocol for human embryonic stem cells using crotonate. Star. Protoc. 2, 100659. 10.1016/j.xpro.2021.100659 34286291 PMC8273405

[B9] Glancy B. (2020). Visualizing mitochondrial form and function within the cell. Trends Mol. Med. 26, 58–70. 10.1016/j.molmed.2019.09.009 31706841 PMC6938546

[B10] Hwang Y. Kwak S. Lee S. Kim H. Lee S. E. Kim J.-H. (2016). Psat1-Dependent fluctuations in α-Ketoglutarate affect the timing of ESC differentiation. Cell. Metab. 24, 494–501. 10.1016/j.cmet.2016.06.014 27476977

[B11] Ishihara N. Nomura M. Jofuku A. Kato H. Suzuki S. O. Masuda K. (2009). Mitochondrial fission factor Drp1 is essential for embryonic development and synapse formation in mice. Nat. Cell. Biol. 11, 958–966. 10.1038/ncb1907 19578372

[B12] Jasra I. T. Cuesta-Gomez N. Verhoeff K. Marfil-Garza B. A. Dadheech N. James Shapiro A. M. (2023). Mitochondrial regulation in human pluripotent stem cells during reprogramming and β cell differentiation. Front. Endocrinol. 14, 1236472. 10.3389/fendo.2023.1236472 PMC10623316 37929027

[B13] Kasahara A. Scorrano L. (2014). Mitochondria: from cell death executioners to regulators of cell differentiation. Trends Cell. Biol. 24, 761–770. 10.1016/j.tcb.2014.08.005 25189346

[B14] Kaspar S. Oertlin C. Szczepanowska K. Kukat A. Senft K. Lucas C. (2021). Adaptation to mitochondrial stress requires CHOP-Directed tuning of ISR. Sci. Adv. 7, eabf0971. 10.1126/sciadv.abf0971 34039602 PMC8153728

[B15] Lisowski P. Kannan P. Mlody B. Prigione A. (2018). Mitochondria and the dynamic control of stem cell homeostasis. EMBO Rep. 19, e45432. 10.15252/embr.201745432 29661859 PMC5934764

[B16] Martínez-Reyes I. Diebold L. P. Kong H. Schieber M. Huang H. Hensley C. T. (2016). TCA cycle and mitochondrial membrane potential are necessary for diverse biological functions. Mol. Cell. 61, 199–209. 10.1016/j.molcel.2015.12.002 26725009 PMC4724312

[B17] Mick E. Titov D. V. Skinner O. S. Sharma R. Jourdain A. A. Mootha V. K. (2020). Distinct mitochondrial defects trigger the integrated stress response depending on the metabolic state of the cell. eLife 9, e49178. 10.7554/eLife.49178 32463360 PMC7255802

[B18] Mitra K. Wunder C. Roysam B. Lin G. Lippincott-Schwartz J. (2009). A hyperfused mitochondrial state achieved at G1–S regulates cyclin E buildup and entry into S phase. Proc. Natl. Acad. Sci. 106, 11960–11965. 10.1073/pnas.0904875106 19617534 PMC2710990

[B19] Nasca A. Legati A. Baruffini E. Nolli C. Moroni I. Ardissone A. (2016). Biallelic mutations in DNM1L are associated with a slowly progressive infantile encephalopathy. Hum. Mutat. 37, 898–903. 10.1002/humu.23033 27328748 PMC5108486

[B20] Oceguera-Yanez F. Kim S. Matsumoto T. Tan G. W. Xiang L. Hatani T. (2016). Engineering the AAVS1 locus for consistent and scalable transgene expression in human iPSCs and their differentiated derivatives. Methods 101, 43–55. 10.1016/j.ymeth.2015.12.012 26707206

[B21] Quirós P. M. Prado M. A. Zamboni N. D’Amico D. Williams R. W. Finley D. (2017). Multi-omics analysis identifies ATF4 as a key regulator of the mitochondrial stress response in mammals. J. Cell. Biol. 216, 2027–2045. 10.1083/jcb.201702058 28566324 PMC5496626

[B22] Rooney J. P. Ryde I. T. Sanders L. H. Howlett E. H. Colton M. D. Germ K. E. (2015). “PCR based determination of mitochondrial DNA copy number in multiple species,” in Mitochondrial regulation: methods and protocols. Editors Palmeira C. M. Rolo A. P. (New York: New York, NY: Springer). 10.1007/978-1-4939-1875-1_3 PMC4312664 25308485

[B23] Ryoo H. D. (2024). The integrated stress response in metabolic adaptation. J. Biol. Chem. 300, 107151. 10.1016/j.jbc.2024.107151 38462161 PMC10998230

[B24] Smirnova E. Griparic L. Shurland D.-L. van der Bliek A. M. (2001). Dynamin-related protein Drp1 is required for mitochondrial division in mammalian cells. Mol. Biol. Cell. 12, 2245–2256. 10.1091/mbc.12.8.2245 11514614 PMC58592

[B25] Steffen J. Ngo J. Wang S.-P. Williams K. Kramer H. F. Ho G. (2022). The mitochondrial fission protein Drp1 in liver is required to mitigate NASH and prevents the activation of the mitochondrial ISR. Mol. Metab. 64, 101566. 10.1016/j.molmet.2022.101566 35940556 PMC9420962

[B26] Tischler J. Gruhn W. H. Reid J. Allgeyer E. Florian B. Marr C. (2019). Metabolic regulation of pluripotency and germ cell fate through α‐ketoglutarate. EMBO J. 38, e99518. 10.15252/embj.201899518 30257965 PMC6315289

[B27] Twig G. Elorza A. Molina A. J. A. Mohamed H. Wikstrom J. D. Walzer G. (2008). Fission and selective fusion govern mitochondrial segregation and elimination by autophagy. EMBO J. 27, 433–446. 10.1038/sj.emboj.7601963 18200046 PMC2234339

[B28] Wakabayashi J. Zhang Z. Wakabayashi N. Tamura Y. Fukaya M. Kensler T. W. (2009). The dynamin-related GTPase Drp1 is required for embryonic and brain development in mice. J. Cell. Biol. 186, 805–816. 10.1083/jcb.200903065 19752021 PMC2753156

[B29] Wang L. Li X. Hanada Y. Hasuzawa N. Moriyama Y. Nomura M. (2021). Dynamin-related protein 1 deficiency accelerates lipopolysaccharide-induced acute liver injury and inflammation in mice. Commun. Biol. 4, 894. 10.1038/s42003-021-02413-6 34290349 PMC8295278

[B30] Wang Y. Marine B. Chen D. (2024). Mitochondrial regulation in stem cells. Trends Cell. Biol. 34, 685–694. 10.1016/j.tcb.2023.10.003 37919163 PMC11193947

[B31] Watanabe K. Ueno M. Kamiya D. Nishiyama A. Matsumura M. Wataya T. (2007). A ROCK inhibitor permits survival of dissociated human embryonic stem cells. Nat. Biotechnol. 25, 681–686. 10.1038/nbt1310 17529971

[B32] Waterham H. R. Koster J. van Roermund C. W. Mooyer P. A. Wanders R. J. Leonard J. V. (2007). A lethal defect of mitochondrial and peroxisomal fission. N. Engl. J. Med. 356, 1736–1741. 10.1056/NEJMoa064436 17460227

[B33] Whitley B. N. Lam C. Cui H. Haude K. Bai R. Escobar L. (2018). Aberrant Drp1-mediated mitochondrial division presents in humans with variable outcomes. Hum. Mol. Genet. 27, 3710–3719. 10.1093/hmg/ddy287 30085106 PMC6196655

[B34] Zebrucka P. Koryga I. Mnich K. Ljujic M. Samali A. Gorman A. M. (2016). The integrated stress response. EMBO Rep. 17, 1374–1395. 10.15252/embr.201642195 27629041 PMC5048378

[B35] Zhang H. Menzies K. J. Auwerx J. (2018). The role of mitochondria in stem cell fate and aging. Development 145, dev143420. 10.1242/dev.143420 29654217 PMC5964648

[B36] Zhong X. Cui P. Cai Y. Wang L. He X. Long P. (2019). Mitochondrial dynamics is critical for the full pluripotency and embryonic developmental potential of pluripotent stem cells. Cell. Metab. 29, 979–992. 10.1016/j.cmet.2018.11.007 30527743

[B37] Zhou G. Meng S. Li Y. Ghebre Y. T. Cooke J. P. (2016). Optimal ROS signaling is critical for nuclear reprogramming. Cell. Rep. 15, 919–925. 10.1016/j.celrep.2016.03.084 27117405 PMC4856580

