# Supplementary material for: Loss of dynamin 1-like protein impairs mitochondrial function and self-renewal, and activates the integrated stress response in human embryonic stem cells
Source: Front Genet. 2025 Jul 28;16:1628178. doi: 10.3389/fgene.2025.1628178 (PMC12336044; doi:10.3389/fgene.2025.1628178)
Supplement: Supplementary file 1 [file DataSheet1.docx]

Supplemental Appendix

Loss of dynamin 1-like protein impairs mitochondrial function and self-renewal and activates integrated stress response in human embryonic stem cells

Artur Cieslar-Pobuda, PhD ^1,2^, Safak Caglayan, PhD ^1, 3^

^1^Centre for Molecular Medicine Norway, University of Oslo and Oslo University Hospital, Oslo, Norway

^2^Department of Cancer Immunology, Institute of Cancer Research, Oslo University Hospital, Oslo, Norway

^3^University Hospital of North Norway, Tromsø, Norway


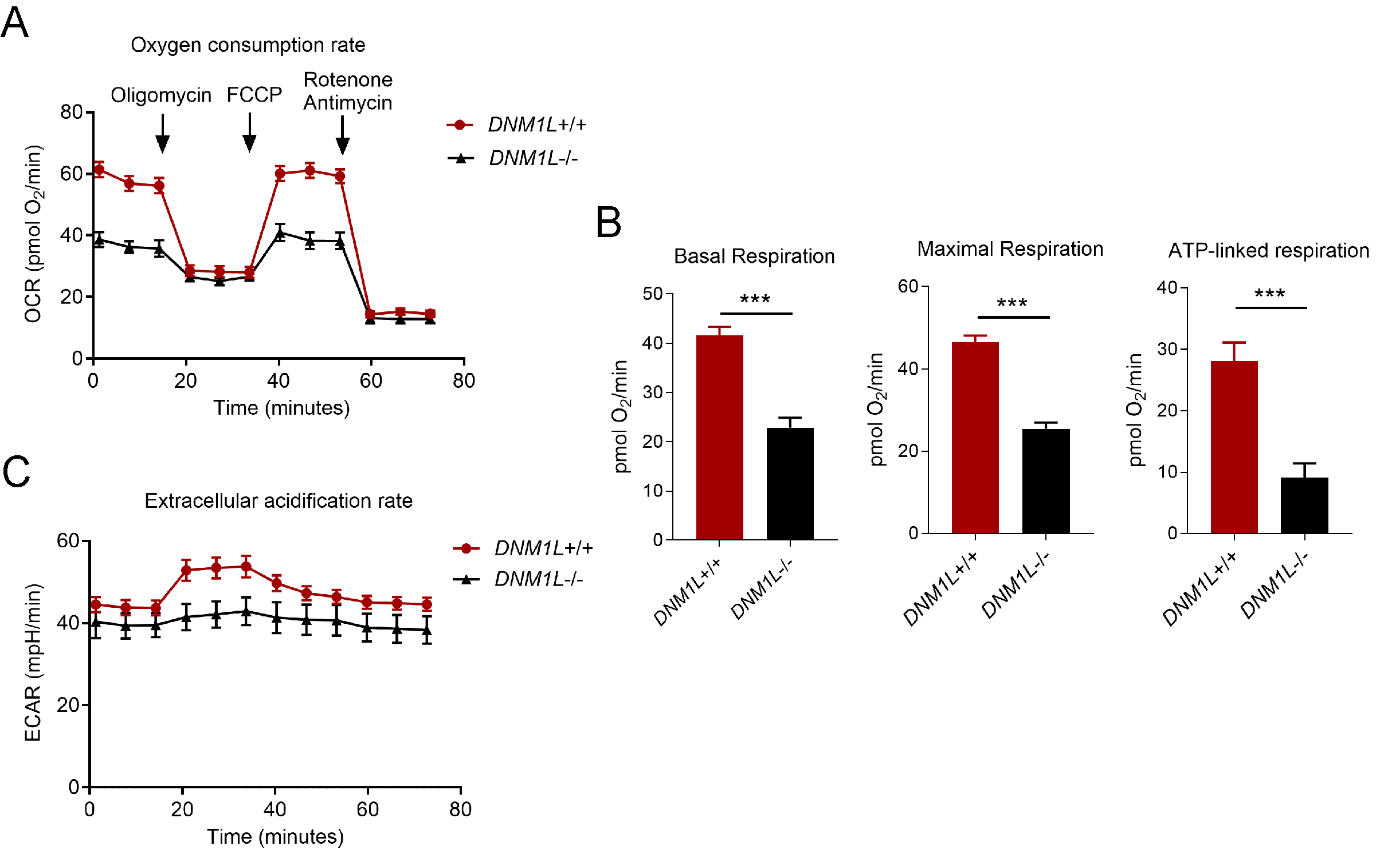


Supplemental Figure S1. Unnormalized oxygen consumption and extracellular acidification rates in *DNM1L*^+/+^ and *DNM1L*^-/-^ hESCs measured by Seahorse Assay.

A) Oxygen consumption rates (OCR) in *DNM1L*^+/+^ and *DNM1L*^-/-^ hESCs. N = 2 experiments. Mean ± SEM is shown.

B) Basal respiration, maximal respiration and ATP-linked respiration in *DNM1L*^+/+^ and *DNM1L*^-/-^ hESCs. N = 2 experiments. Mean ± SEM is shown. Student’s t-test is used to analyze differences between two groups, *** p < 0.001.

C) Extracellular acidification rates (ECAR) in *DNM1L*^+/+^ and *DNM1L*^-/-^ hESCs. N = 2 experiments. Mean ± SEM is shown.


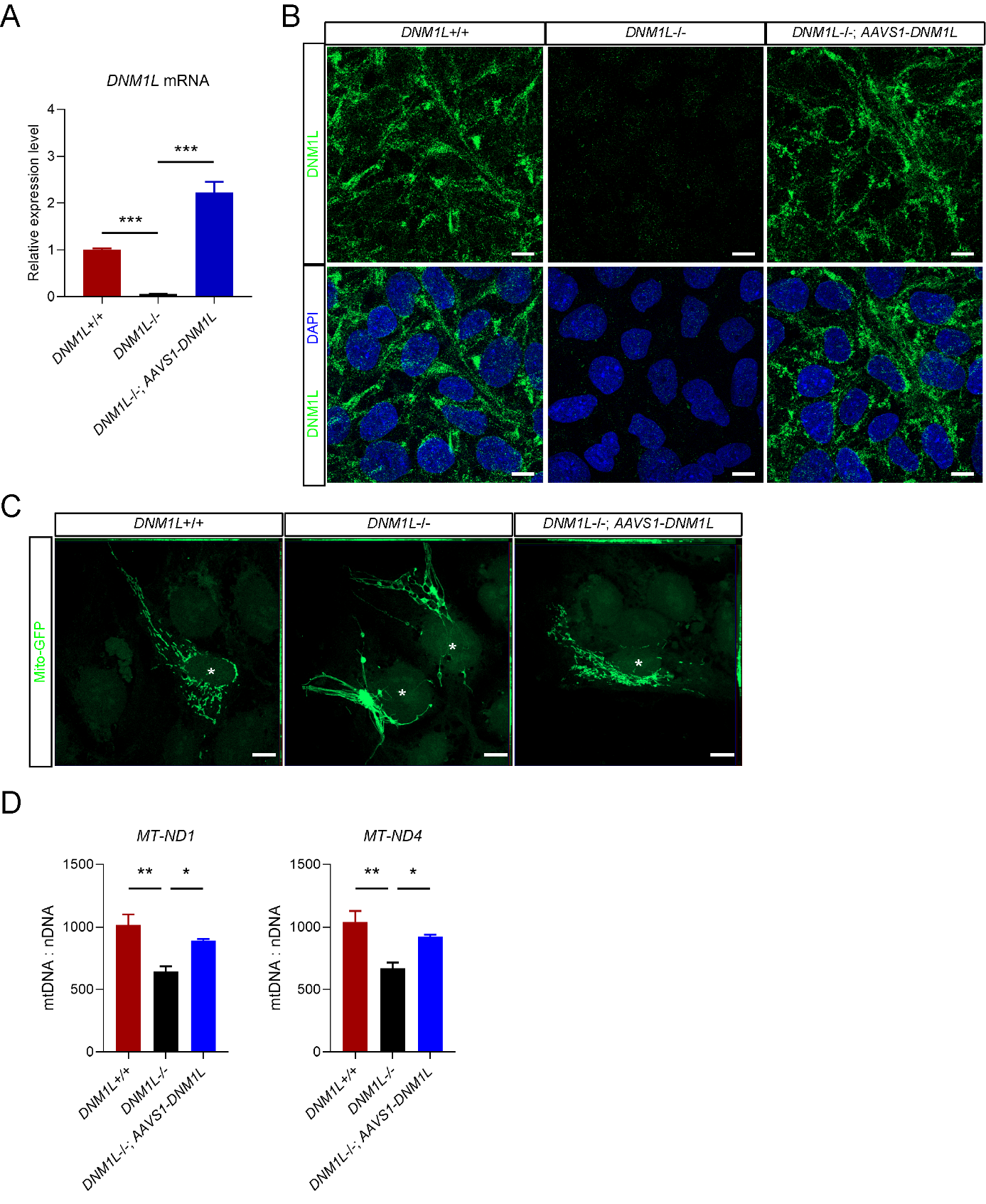


Supplemental Figure S2. Re-expression of DNM1L rescues mitochondrial morphology and DNA content in DNM1L deficient hESCs.

A) QRT-PCR analysis of *DNM1L* mRNA expression in *DNM1L*^+/+^, *DNM1L*^-/-^, and *DNM1L* rescue hESCs. Gene expression is normalized to the expression of 18S control gene and presented as relative to gene expression levels in *DNM1L*^+/+^ hESCs. N = 3 experiments. Mean ± SEM is shown. Analysis of variance (ANOVA) with Sidak post hoc test is used to analyze differences between the groups, *** p < 0.001.

B) Immunostaining for DNM1L in *DNM1L*^+/+^, *DNM1L*^-/-^, and *DNM1L* rescue hESCs. DAPI is used to stain nuclei. Scale bar, 10 µM.

C) Representative confocal microscopy pictures of *DNM1L*^+/+^, *DNM1L*^-/-^, and *DNM1L* rescue hESCs transduced with mito-GFP. Scale bar, 10 µM.

D) Mitochondrial DNA content in *DNM1L*^+/+^, *DNM1L*^-/-^, and *DNM1L* rescue hESCs. Copy number of mitochondrial *ND1* and *ND4* genes are measured using qPCR, and normalized to nuclear *B2M* gene. N = 3 experiments. Analysis of variance (ANOVA) with Sidak post hoc test is used to analyze differences between the groups. * p < 0.05, ** p < 0.01.


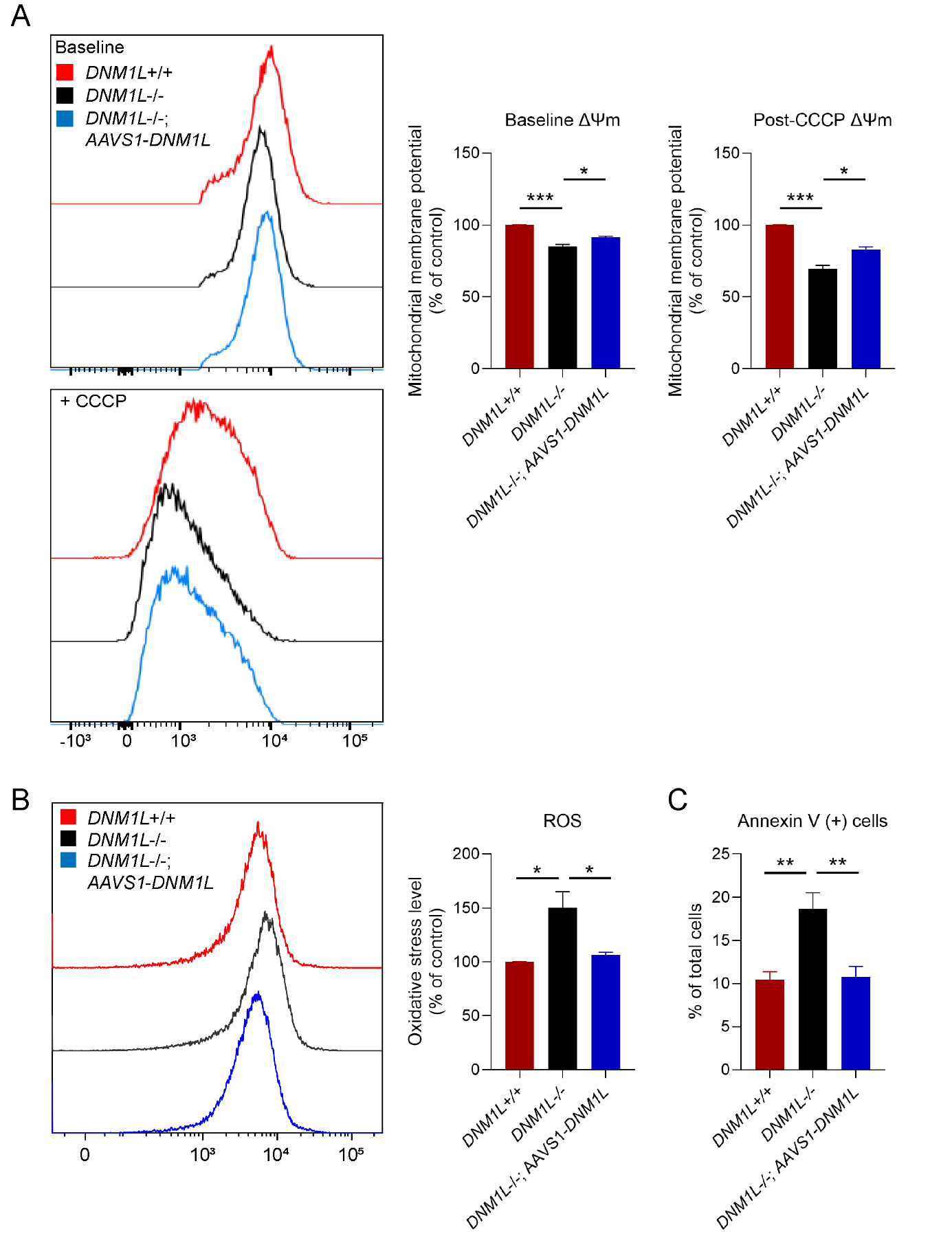


Supplemental Figure S3. Re-expression of DNM1L rescues mitochondrial membrane potential and reduces oxidative stress and apoptosis in DNM1L deficient hESCs.

A) Flow cytometry measurements of mitochondrial membrane potential in *DNM1L*^+/+^, *DNM1L*^-/-^, and *DNM1L* rescue hESCs. CCCP is used to depolarize the mitochondrial membranes. N = 4 experiments. Mean ± SEM is shown.

B) Flow cytometry measurements of reactive oxygen species (ROS) in *DNM1L*^+/+^, *DNM1L*^-/-^, and *DNM1L* rescue hESCs labeled with oxidative stress indicator CM-H2DCFDA. Mean fluorescence intensity values are normalized to the values of labeled *DNM1L^+/+^* hESCs. N = 4 experiments. N = 4 experiments.

C) Quantification of flow cytometry measurements of basal level of apoptosis in *DNM1L*^+/+^, *DNM1L*^-/-^, and *DNM1L* rescue hESCs. N = 5 experiments.


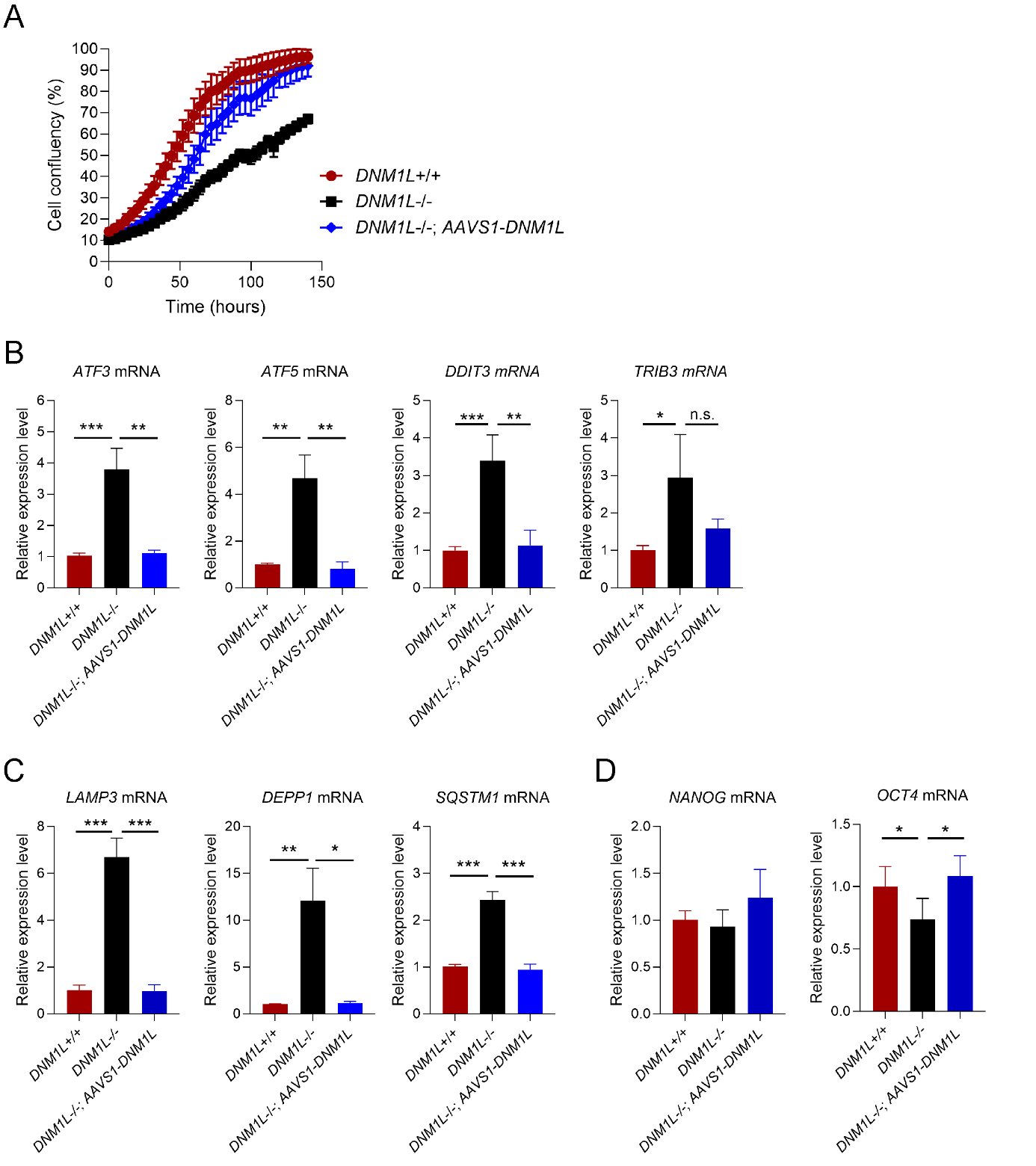


Supplemental Figure S4. Re-expression of DNM1L restores self-renewal, attenuates integrated stress response, and rescues OCT4 expression in DNM1L deficient hESCs.

A) Confluency of *DNM1L*^+/+^, *DNM1L*^-/-^ and *DNM1L* rescue hESCs over time assessed by Incuyte instrument. N = 4 experiments.

B) QRT-PCR analysis of stress response genes *ATF3, ATF5, DDIT3 and TRIB3* in *DNM1L*^+/+^, *DNM1L*^-/-^ and *DNM1L* rescue hESCs. N = 4 experiments. Mean ± SEM is shown.

C) QRT-PCR analysis of selective autophagy genes *LAMP3, DEPP1* and *SQSTM1 (p62)* in *DNM1L*^+/+^, *DNM1L*^-/-^ and *DNM1L* rescue hESCs. N = 4 experiments. Mean ± SEM is shown.

D) QRT-PCR analysis of *NANOG* and *OCT4* mRNA in *DNM1L*^+/+^, *DNM1L*^-/-^ and *DNM1L* rescue hESCs. N = 4 experiments. Mean ± SEM is shown. Analysis of variance (ANOVA) with Sidak post hoc test is used to analyze differences between the groups. n.s. not significant, * p < 0.05, ** p < 0.01, *** p < 0.001.

Supplemental Table S1. Primer sequences.

| Primer name | Sequence (5′ → 3′) |
| --- | --- |
| DNM1L sgRNA 1 | TCATTGCCTTTCAGATGCGG |
| DNM1L sgRNA 2 | AAGTGGATCAACAGATTCTA |
| DNA183 | CTCAGGTTCTGGGAGAGGGTAG |
| DNA803 | TCGACTTCCCCTCTTCCGATG |
| DNA804 | GAGCCTAGGGCCGGGATTCTC |
| 18S Forward | CTACCACATCCAAGGAAGGCA |
| 18S Reverse | TTTTTCGTCACTACCTCCCCG |
| DNM1L Forward | TTGGTGGGTGACCTGTCTCT |
| DNM1L Reverse | TGCTCCTTGCTTGTTTCTCC |
| OCT4 Forward | CATCTCACGGTTCCTGGAGT |
| OCT4 Reverse | GGTAGGTTGCCCCAGAAGTA |
| NANOG Forward | CATGAGTGTGGATCCAGCTTG |
| NANOG Reverse | CCTGAATAAGCAGATCCATGG |
| DLK1 Forward | CCCGAGTTCACAGGTCTCAC |
| DLK1 Reverse | GGTAGGCCAGCCCATAGC |
| RAX Forward | CGTTCGAGAAGTCCCACTACC |
| RAX Reverse | ATGGAGGACACTTCCAGCTTC |
| LHX2 Forward | TGTTTCAGCAAGGACGGTAG |
| LHX2 Reverse | TGATAAACCAAGTCCCGAGC |
| MSX1 Forward | GAGATCAGCGTTGGAGAGTCC |
| MSX1 Reverse | GGAGAACCAGGAGATGTCTTTATCTT |
| HAND1 Forward | CGCAGAAGGGTTAAACAGGT |
| HAND1 Reverse | CGGGCAAGGCTGAAAATGAG |
| CDX2 Forward | ATCACCATCCGGAGGAAAG |
| CDX2 Reverse | TGCGGTTCTGAAACCAGATT |
| SOX17 Forward | GGTGTGAATCTCCCCGACAG |
| SOX17 Reverse | TAATATACCGCGGAGCTGGC |
| GATA4 Forward | AGCTCCGTGTCCCAGACG |
| GATA4 Reverse | TCTGTGGAGACTGGCTGACG |
| GATA6 Forward | TGTCGAGCTGGGAGGACTT |
| GATA6 Reverse | GCGAGGGTCTGGTACATCTC |
| MT-ND1 Forward | ACGCCATAAAACTCTTCACCAAAG |
| MT-ND1 Reverse | GGGTTCATAGTAGAAGAGCGATGG |
| MT-ND4 Forward | ACCTTGGCTATCATCACCCGAT |
| MT-ND4 Reverse | AGTGCGATGAGTAGGGGAAGG |
| Nuclear-B2M Forward | CACTGAAAAAGATGAGTATGCC |
| Nuclear-B2M-Reverse | AACATTCCCTGACAATCCC |
| p62/SQSTM1 Forward | AATCAGCCTCTGGTCCATCG |
| p62/SQSTM1 Reverse | TTCTTTTCCCTCCGTGCTC |
| TRIB3 Forward | CCGTCTTGGGCCCTATGT |
| TRIB3 Reverse | CTTCCTGGACGGGGTACA |
| DDIT3/CHOP Forward | CCTCCTGGAAATGAAGAGGA |
| DDIT3/CHOP Forward | CTCTGGGAGGTGCTTGTGAC |
| ATF3 Forward | CCATCACAAAAGCCGAGGTAG |
| ATF3 Reverse | TCACACTTTCCAGCTTCTCC |
| ATF5 Forward | GCTCGTAGACTATGGGAAACTC |
| ATF5 Reverse | AATCAACTCGCTCAGTCATCC |
| LAMP3 Forward | TCAGCCATCGTCAGTCAAGA |
| LAMP3 Reverse | TGAAGTATCTCCGAGGTGAAAAA |
| DEPP1 Forward | CCTGCTCATCCATTCTCCTG |
| DEPP1 Reverse | CGTGGTCTCCCGAATTGTG |

Supplemental Table S2. List of the antibodies used in the study.

| Antibody (Clone) | Company | Catalog # |
| --- | --- | --- |
| Goat anti-beta Actin (C4) | Santa Cruz | SC-1615 |
| Rabbit anti-NANOG (D73G4) | Cell Signaling Technologies | 4903S |
| Mouse anti-OCT3/4 (C-10) | Santa Cruz | SC-5279 |
| Rabbit anti-DNM1L (D6C7) | Cell Signaling Technologies | 8570S |
| Rabbit anti-LC3 | Cell Signaling Technologies | 12741T |
| Rabbit anti-ATG12 | Cell Signaling Technologies | 4180T |
| Rabbit anti-ATG 5 | Cell Signaling Technologies | 12994T |
| Mouse anti-p62/SQSTM1 | Cell Signaling Technologies | 88588S |
| Rabbit anti-ATF4 | Cell Signaling Technologies | 11815S |
| Rabbit P-eIF2lpha | Cell Signaling Technologies | 3398T |
| Rabbit eIF2alpha | Cell Signaling Technologies | 5324T |
| Rabbit anti BIP | Cell Signaling Technologies | 3183S |
| Alexa-488 Goat anti-rabbit IgG | Thermo Fisher Scientific | A11034 |
| Alexa-546 Donkey anti-mouse IgG | Thermo Fisher Scientific | A10036 |
| Alexa-546 Donkey anti-rabbit IgG | Thermo Fisher Scientific | A10040 |
| HRP Goat anti-mouse IgG | Jackson Immunoresearch | 115-035-146 |
| HRP Goat anti-rabbit IgG | Jackson Immunoresearch | 111-035-144 |
